# Supplementary material for: A Framework for Effective Application of Machine Learning to Microbiome-Based Classification Problems
Source: mBio. 2020 Jun 9;11(3):e00434-20. doi: 10.1128/mBio.00434-20 (PMC7373189; doi:10.1128/mBio.00434-20)
Supplement: TABLE S1 [file mBio.00434-20-st001.pdf]

Table S1. An aspirational rubric for evaluating the rigor of ML practices applied to microbiome data.

Practice	Poor	Good	Better
Source of data	Data do not reflect intended application (e.g., data pertain to only patients with carcinomas but model is expected to predict advanced adenomas).	Data are appropriate for intended application.	Data reflect intended use and will persist (e.g., same OTU assignments for new fecal samples).
Study cohort	Test data resampled to remove class imbalance (e.g., test data resampled to have an equal number of patients with carcinomas as patients with healthy colons, which does not reflect reality.)	Test data are reflective of the population to which the model will be applied.	Model tested on multiple cohorts with potentially different class balances.
Model selection	No justification for classification method.	Model choice is justified for intended application.	Different modeling choices (justified for intended application) are tested.
Model development	No hyperparameter tuning.	Different hyperparameter settings are explored on training data.	Hyperparameter grid search performed by cross-validation on the training set.
Model evaluation	Performance reported on the data used to train the model.	Performance reported on held-out test data.	Performance reported on multiple held-out test sets.
Evaluation metrics	Reported performance according to a metric that is not appropriate for intended application (e.g., when predicting rare outcome, accuracy metric is not reliable).	Reported performance in terms of a metric that is appropriate for intended application and includes confidence intervals.	Reported multiple metrics with confidence intervals.
Model interpretation	No model interpretation.	Follow-up analyses to determine what is driving model performance.	Hypotheses based on feature importances are generated and tested.
